# What Predicts a Longer Period of Pain in Patients Referred to an Interdisciplinary Center for Pain Care?

**DOI:** 10.3390/ijerph21070845

**Published:** 2024-06-28

**Authors:** Helen Cristina Nogueira Carrer, Melina Nevoeiro Haik, Gabriela Espósito, Fernando Augusto Vasilceac, Cristiane de Sousa Melo, Maria Gabriela Pedroso, Karina Gramani Say

**Affiliations:** 1 Department of Physical Therapy, Federal University of São Carlos (UFSCar), Interdisciplinary Center for Pain Care at UFSCar, São Carlos 13565-905, Brazil; melinahaik@ufscar.br (M.N.H.); cristianemelo@estudante.ufscar.br (C.d.S.M.); gabriela.pedroso@gmail.com (M.G.P.); 2 Department of Gerontology, Federal University of São Carlos (UFSCar), Interdisciplinary Center for Pain Care at UFSCar, São Carlos 13565-905, Brazil; gabrielaesposito@estudante.ufscar.br (G.E.); fervasilceac@ufscar.br (F.A.V.);

**Keywords:** chronic pain, biopsychosocial model, interdisciplinary care, health management

## Abstract

Chronic musculoskeletal pain (CMP) is a global health condition that affects thousands of people. CMP can substantially affect the functional capacity and quality of life of the people impacted, resulting in high costs for health care and social security systems. Sociodemographic factors may play a significant role in pain chronification prevention and control programs. Thus, current risk factors for CMP must be seriously considered as part of an interdisciplinary management strategy. The purpose of the study was to identify the primary sociodemographic characteristics of CMP patients at a multidisciplinary and specialized center for chronic pain. This is a retrospective investigation based on a review of medical records. Age, gender, income, and the time of onset of pain symptoms were among the variables included in the analyzed data. To analyze variables related to the duration of discomfort, a multiple regression model was utilized. Sociodemographic factors explained 37.94% of experiencing prolonged pain, according to the study’s findings. Being female and having a family income above the minimum wage were variables that were directly proportional to discomfort duration. Age was not associated with a prolonged duration of pain perception.

## 1. Introduction

Chronic musculoskeletal pain (CMP) refers to persistent discomfort that originates from musculoskeletal structures, including bones and joints [1]. CMP has been identified as the main global indicator of morbidity [2,3,4,5]. According to the Academy of Pain Medicine, pain symptoms affect more people than cardiovascular disease, cancer, and diabetes [6,7]. An understanding of CMP and its implications for physical disability and impaired performance in life activities is necessary for planning strategies, prioritizing care, and promoting better functionality.

According to a recent analysis of the Global Burden of Disease (GBD) 2019 data, an estimated 1.71 billion individuals worldwide are affected by musculoskeletal disorders [8]. The prevalence of CMP in Brazil in 2021 corresponds to 45.59% of the entire population [9]. High-income countries see the greatest impact in terms of population, with a total of 441 million individuals affected [8]. In fact, little is known about the prevalence of CMP in the general population in developing countries. Several studies on the epidemiology of CMP in the general population have utilized non-representative samples that may not accurately reflect the population with better access to healthcare services [10].

The functional limitations associated with CMP may lead to socioeconomic issues for patients, as well as for the health care and social security systems. This relationship can occur since CMP is connected to higher care costs and an underlying increased risk of early absenteeism and disability [4,11]. Its socioeconomic repercussions are an encouragement for a reorganization of care delivery [12]. CMP is a prominent contributor to the overall burden of disease, resulting in 139 million disability-adjusted life years (DALYs) [13]. The COVID-19 pandemic has exacerbated these individuals’ suffering, with the expectation that the severity of this issue will continue to increase above pre-pandemic levels [14].

The chronicity of musculoskeletal pain, which is characterized by the presence of pain perception lasting longer than three months, may be attributed to variables beyond those generated by physical circumstances [15,16,17]. People experiencing socioeconomic vulnerability, such as those found in low- and middle-income countries, encounter psychosocial barriers that can worsen pain symptoms and restrict their level of functioning [18,19,20,21,22,23]. The occurrence of chronic pain in high-income nations varies between 19.0% and 20.4% [21,22]. On the other hand, in poor and middle-income countries, it can impact up to 26% of the general population and 86% of workers [23]. Indicators of a lack of financial resources and instability are often linked to symptoms of CMP perception.

Mental health can influence the perception of CMP [24]. Anxiety and depression are associated with increased pain responses in CMP. Fatigue and sleeping problems can further exacerbate this perception of pain, creating a cycle where pain leads to sleep disturbances and fatigue, which in turn worsen the perception of pain [25]. This intricate relationship between mental health, sleep disturbances, and pain perception underscores the importance of adopting a holistic approach to managing chronic pain, which includes addressing mental health issues and improving sleep quality alongside traditional pain management strategies [26].

Despite its significant impact on various health-related aspects, education is frequently overlooked as a crucial socioeconomic predictor of health. The extensive significance of health makes it the most influential factor in determining a person’s well-being [27]. The amount of formal education is highly positively connected with factors such as life expectancy, illness, and health-related behavior [28]. Individuals with a greater level of education generally report better health and physical functioning compared to those with lower levels of education [27].

Higher education was associated with decreased musculoskeletal pain and physical function limitations, according to a Danish longitudinal study [29]. Nevertheless, prior research has identified limitations regarding the relationship between the level of education and the severity of CMP. Instances of bias in this relationship can be observed in pain sites, the majority with back pain [30], among a restricted population involving mostly the elderly [31,32], and in studies conducted exclusively in developed countries. Comprehending the association between CMP and educational attainment is important for providing information to politicians and taking action.

Statistically relevant sociodemographic evidence is provided by the fact that women are more likely to experience chronic musculoskeletal pain (CMP) [9,33,34]. Literature indicates that women experience more pain than males, which appears to be mediated not only by biological, genetic, and hormonal factors but also by social and behavioral experiences [35,36,37]. The physiological process of aging has also been linked to an increase in the prevalence of CMP among the elderly [38]. However, a report by the National Center for Health Statistics (2006) found a decline in CMP with advancing age, bringing inconsistency to the notion that the prevalence of pain increases over aging [39].

Although sex, educational level, and socioeconomic conditions are important variables associated with chronic pain, structural racism exposes black men and black women to a higher susceptibility to disease [40,41]. However, the findings of the Health and Retirement Study, which included persons in the United States who were over the age of 50, did not reveal any significant differences in pain-related impairment based on race or ethnicity [42]. Oliveira et al. (2023) demonstrated that race or skin color is a contributing factor to chronic pain in older individuals [43]. Additional research is required to investigate the relationship between race, skin color, and CMP according to the disparity between existing literature and the limited studies conducted on the older population.

The current recommendation emphasizes the importance of incorporating biopsychosocial factors into the assessment of individuals diagnosed with CMP, as well as the utilization of interdisciplinary approaches in their treatment. In order to optimize the implementation of these assessments and interventions, it is imperative to enhance the strategizing of patient profile screening [44]. As a result, it is imperative to comprehend the characteristics of those impacted by CMP in order to develop appropriate therapy [45]. Unfortunately, a significant proportion of current rehabilitation interventions continue to be diffused across the domains of social assistance and healthcare [46]. In order to address inequalities and ensure fairness in pain evaluation and treatment, it is recommended to focus on enhancing comprehension of the sociodemographic characteristics. This approach can effectively enhance the accessibility of pain management services and facilitate better communication between patients and healthcare providers [47].

Chronic pain services, on the other hand, are confronted with substantial limits, particularly within public healthcare institutions. In many cases, these limits are the result of a lack of trained staff or are a consequence of the physical and organizational constraints that are inherent to the services themselves. In spite of the progress that has been made, the development of integrative and specialized pain care continues to face a great deal of resistance [48,49]. The Interdisciplinary Center for Pain Care at the Federal University of Sao Carlos is a specialized service that focuses on addressing the complex needs of patients with chronic pain. Established with the goal of delivering comprehensive and interdisciplinary treatment, the sociodemographic characteristics have been evaluated as an innovative strategy for connecting health and social assistance.

The aim of this study is to identify sociodemographic characteristics associated with the duration of pain experienced by patients with CMP who had either attended or were on the waiting list at an interdisciplinary reference center. The study hypothesizes that sociodemographic characteristics such as older age, female gender, black race, and low income contribute to the prolonged duration of CMP. The evaluated variables aid in understanding unequal pain and could aid in managing and preventing chronic pain in connection with improving equitable access to therapies [50].

## 2. Materials and Methods

This is a cross-sectional and retrospective study that analyzed the medical records and screening profiles of chronic pain patients who sought treatment at the Interdisciplinary Center for Pain Care of the Federal University of Sao Carlos. To reduce the amount of personal information belonging to individuals, just one researcher examined clinical records from March to December 2020.

The medical records were chosen based on the following criteria: the patient had musculoskeletal pain, had complained of discomfort for more than three months, was over 18 years old, and had already sought medical treatment for rehabilitation. The ethics committee for human subject research at the Federal University of So Carlos approved the study (Process No. 27744920.4.0000.5504). All individuals evaluated signed the informed consent form.

The Interdisciplinary Center for Pain Care of the Federal University of Sao Carlos has an extensive and specific assessment protocol for chronic pain that includes questions such as clinical history, pain chronology, pain intensity (visual analog scale), the severity of pain, and its impacts on functioning (Brief Pain Inventory). In the case of multiple pains, the worst pain was chosen to be included in the study.

Demographic data were collected during the first interviews. In relation to the patient’s gender/sex, they could choose male or female options. The patient’s race was self-declared as black, brown, or white. The variable educational attainment data were collected using the following categories: illiterate, completed elementary school, completed high school, or completed university study. The patient’s income was self-disclosed and classified according to the criterion variable of minimal salary thresholds: less than 1, between 1 and 2, between 3 and 5, and greater than 5.

The variable to be explained (the dependent variable) is the self-reported years of chronic pain duration. Age, gender, education, income, and race were the sociodemographic variables collected. Age, gender, and income were included in the regression model as independent explanatory variables (predictors) due to their stronger correlations with pain duration. The variables that explain the duration of chronic pain in the investigated sample are described and classified in Table 1.

The description of the sample was made using mean and standard deviation measurements for numerical variables and percentages for categorical variables. A multiple linear regression model estimated by the ordinary least squares (OLS) method was adopted to analyze correlates of pain duration. The use of multiple linear regression models to identify the determinants of the time of illness of patients is common in hospital contexts [50]. Thus, the model assumes the form of a log-linear model, and the results of the coefficients associated with the explanatory variables can be interpreted as coefficients of elasticity. The mathematical expression that represents the model is as follows:

Ci = β^′ Xi + ε(1)

Ci is the duration of chronic pain;
Xi is the set of explanatory variables used to explain the duration of chronic pain reported by patients;
β is the set of estimated coefficients for each predictor variable;
ε is the residual error of the model.

All analyses were conducted with the SPSS (Statistical Package for the Social Sciences, version 20) software and a significance level of 0.05 (5%). The coefficients of each independent variable, their confidence intervals, standard error, and *p* values were used to present the model’s results.

## 3. Results

The evaluation of 78 medical records and screening forms included data from 52 individuals. The descriptive statistics for sociodemographic variables are provided in Table 2. The patients’ ages ranged from 19 to 79, with a mean of 54 and a standard deviation of 17 years. 89% of the subjects reported receiving less than five minimum wages (Table 2).

Sixty-seven percent of the participants reported experiencing pain for one to ten years, with pain persisting varied from six months to twenty-five years (mean: 4 ± 5.70 years).

The proposed regression model is useful for estimating the duration of pain in relation to sociodemographic variables, as indicated by the F test *p*-value of 0.001 and the significance of only gender and income being related to the duration of pain. These variables accounted for 37.94% of the variance in pain duration (R^2^ = 0.3794).

Table 3 presents the results of the regression analysis. In the proposed model, the coefficient for the sex variable showed that women reported experiencing pain for significantly more time than men did (*p* = 0.003). The income variable’s coefficient was likewise statistically significant (*p* = 0.002), suggesting that higher income was associated with prolonged pain duration.

## 4. Discussion

This retrospective cross-sectional study investigated the contribution of socio-demographic characteristics such as age, gender, and income as potential variables relating to the duration of musculoskeletal pain in patients assisted by a public service specializing in CMP. In 37.94% of cases, the results indicate that the probability of prolonged pain was explained by sex and income. The variables that were directly proportional to the duration of pain were being female and having a family income greater than twice the minimum wage. The gender indicator was supported by the hypothesis of this research. The income indicator contradicts the initial hypothesis, which predicted an inversely proportional relationship between pain chronicity and income. The hypothesis that older age was associated with prolonged duration of CMP was refuted, considering our sample.

The need to maintain functional capacity as the world’s population ages increasingly is a major public health issue, especially in low-income countries [51]. The impacts of CMP on the elderly may slow progress regarding a longer, healthier, and more independent life expectancy [52,53]. However, the present study did not find a significant correlation between a longer course of CMP and increasing age. In contrast, the evaluated regression model identified a negative value for the age coefficient, indicating an inverse correlation between the variables of pain duration and age. The meta-analysis conducted by Lautenbacher et al. (2017) suggests that the elderly have higher pain thresholds, a fact that could have implications for the results obtained [54]. On the other hand, Santiago et al. (2023) indicated that the presence of unskilled and non-specialized services results in a significant number of older patients suffering from chronic pain not receiving therapy that is adequate for their condition [55]. Therefore, our finding of a greater proportion of older people requesting a specialized service does not provide an explanation as to whether the aged really develop a higher threshold for pain perception or if there is a lack of support for those who are most susceptible.

Women are significantly more likely than men to suffer from musculoskeletal disorders that culminate in chronic pain. Consequently, the prevalence of CMP is significantly higher in women [56]. Although social and psychological factors have been shown to influence differences in the prevalence and incidence of CMP [57,58], biological differences also mostly explain these results. In women, neuroendocrine interactions mediated by sex support the development and maintenance of increased pain sensitivity and chronicity [59,60]. In addition, women have more resistance fibers than strength fibers and a reduced cross-sectional area of specific muscles, such as the upper trapezius [51]. Consequently, an increase in pain perception and a decline in functional capacity could be responsible for the prolonged duration of symptoms in women. The number of female and male patients was not homogeneous in the present study, precluding a direct correlation analysis between the determining factors for pain and sex. However, the results obtained in the regression analysis identified the male sex as a protective factor for severe pain compared to the female sex. In light of the disparity in the incidence of CMP between the sexes, studies on health management, which are currently in short supply, are essential for planning actions for the prevention, control, and treatment of CMP.

The presence of economic difficulties is closely linked to socioeconomic status, representing a personal perception and an important determinant of disability and psychological suffering. Low income, therefore, acts as a risk factor for the development of pain and, at the same time, can be negatively influenced by pain, which is a bidirectional relationship [26,61,62]. However, in this study, the majority of participants reported an income between three and five salaries. Financial issues and limited income are common challenges to obtaining health care [63]. The main factors contributing to the inability of low-income households to get healthcare are high transportation expenses and a lack of health insurance [64]. Consequently, it is essential that we recognize that multiple factors contribute to the loss of function of a variety of health conditions. Instead of focusing only on the individual’s medical history, it is worthwhile to assess their subjective sense of financial well-being and sense of timely adherence to any form of intervention [65,66]. Responsible professionals must consider access and the user’s ability to maintain use in order to identify financial barriers to adherence, even in the context of drug interventions.

The study’s limitations in generalizing the findings can be attributed to the small sample size and reliance on self-reported data collection. In addition, the model explained only 38% of the variability in pain duration, indicating that other factors, whether intrinsic or extrinsic to the subject, may also contribute significantly to the duration of CMP. However, because it is a study for the planning of health actions, it is intended that the results will provide warnings about the profile of individuals affected by chronic pain and thus motivate preventive, rehabilitation, and policy formation actions and policies for early pain management.

## 5. Conclusions

Several factors may contribute to CMP. Therefore, the guidelines recommend an individualized, multimodal, and interprofessional approach. The most effective strategy for pain treatment and management is planning a care paradigm in primary care, beginning with less specialized care. This study investigates sociodemographic aspects of the local CMP population. Our sample suggests early intervention for chronic pain control and prevention for women and adults with a family income of up to two minimum wages.

## Figures and Tables

**Table 1:** Classification and description of variables associated with chronic pain duration.

Variable	Classification	Variable Description
Age	Numerical	Patient’s age
SEX	Dummy (1 = male; 2 = female)	Male or female; categorical qualitative variable with two separate groups.
INCOME	Ordinal quantitative scale in MW (1 = without income; 2 = up to 1 MW; 3 = 2–5 MW; 4 = above 5 MW)	The monthly value is based on the total individual incomes of household members, which represents the family income.

MW: minimal wages.

**Table 2:** Descriptive statistics: sociodemographic characteristics of the sample.

Variable	Frequency (Percentage)
Sex	
Male	20 (44%)
Female	25 (56%)
Age (years)	
18–35	7 (13.46%)
35–60	21 (40.38%)
Over 60	24 (46.15%)
Race	
White	35 (67%)
Black	5 (9%)
Brown	12 (24%)
Education	
Illiterate	2 (4%)
Elementary School	28 (53.85%)
High school	8 (15.38%)
University education	14 (26.92%)
ICD *	
Spine	32 (62%)
Upper limbs	13 (24%)
Lower limbs	7 (14%)
Income (in the minimum wage)	
up to 1	7 (13.46%)
between 1 and 2	17 (32.69%)
between 3 and 5	23 (44.23%)
above 5	5 (9.62%)

* ICD: International Statistical Classification of Diseases and Related Health Problems.

**Table 3:** Multiple regression analysis between the duration of chronic pain and age, sex, and income of patients.

R Squared	0.37				
Predictors	*β*	CI 95%		Standard Error	*p*-Value
		LI	LS		
Age	−0.016	−0.1051541	0.071611866	0.043957758	0.704
Sex	4.09	1.31013351	6.873407725	1.383462161	0.004 *
Income	2.80	1.13245022	4.481012182	0.832712641	0.001 *

*β* Regression coefficient; CI 95% Confidence Interval; * Significant *p*-value.

## Data Availability

The data presented in this study are available on request from the corresponding author.
